# Making the head: Caspases in life and death

**DOI:** 10.3389/fcell.2022.1075751

**Published:** 2023-01-13

**Authors:** Eva Svandova, Herve Lesot, Paul Sharpe, Eva Matalova

**Affiliations:** ^1^ Faculty of Medicine, Masaryk University, Brno, Czechia; ^2^ Institute of Animal Physiology and Genetics, Czech Academy of Sciences, Brno, Czechia; ^3^ Centre for Craniofacial and Regenerative Biology, Faculty of Dentistry, Oral, and Craniofacial Sciences, King’s College London, London, United Kingdom; ^4^ Department of Physiology, University of Veterinary Sciences, Brno, Czechia

**Keywords:** caspases, development, head, apoptotic, non-apoptotic

## Abstract

The term apoptosis, as a way of programmed cell death, was coined a half century ago and since its discovery the process has been extensively investigated. The anatomy and physiology of the head are complex and thus apoptosis has mostly been followed in separate structures, tissues or cell types. This review aims to provide a comprehensive overview of recent knowledge concerning apoptosis-related molecules involved in the development of structures of head with a particular focus on caspases, cysteine proteases having a key position in apoptotic pathways. Since many classical apoptosis-related molecules, including caspases, are emerging in several non-apoptotic processes, these were also considered. The largest organ of the head region is the brain and its development has been extensively investigated, including the roles of apoptosis and related molecules. Neurogenesis research also includes sensory organs such as the eye and ear, efferent nervous system and associated muscles and glands. Caspases have been also associated with normal function of the skin and hair follicles. Regarding mineralised tissues within craniofacial morphogenesis, apoptosis in bones has been of interest along with palate fusion and tooth development. Finally, the role of apoptosis and caspases in angiogenesis, necessary for any tissue/organ development and maintenance/homeostasis, are discussed. Additionally, this review points to abnormalities of development resulting from improper expression/activation of apoptosis-related molecules.

## Introduction

The head represents the most complex part of the body, encompassing functionally, structurally, and developmentally diverse components. The formation of the cranial region starts during early embryonic periods with the establishment of pharyngeal arches that are populated by neural crest cells. Craniofacial development requires integration of different cellular processes including cell proliferation, differentiation, migration and cell death. The accurate balance between the aforementioned processes, their localisation and precise timing are required for the co-ordination of head development. Recent research, performed mostly in mouse models, shows that caspases are multifunctional enzymes that make contributions to various developmental processes. Abnormalities of craniofacial development resulting from inappropriate expression/activation of caspases and related molecules are discussed in the following chapters.

## Diverse members of the same family

The caspase family contains evolutionary-conserved proteases involved in apoptotic intracellular machinery (Kumar, 2007; Mazzoni and Falcone, 2008). The term “caspases” stands for cysteine-dependent aspartate specific proteases and refers to their ability to specifically recognise and cleave substrates (Stennicke and Salvesen, 1999). The categorisation of caspases reflects their function and structure as shown in Table 1. Persisting inconsistency in caspase classification points to their multiple and unknown activities. In mice there are three major groups of caspases: apoptotic initiators (caspase-8, -9, -2), apoptotic executors (caspase-3, -6, -7), and inflammatory caspases (caspase-1, -11, -12). Caspase-14 having neither relation with apoptosis nor with inflammation, stands alone (Shalini et al., 2015). Some studies exclude caspase-2 from apoptotic initiators to establish an extra group associated with the cell cycle (Van Opdenbosch and Lamkanfi, 2019). Indeed, caspase-2 was demonstrated to cleave MDM2, a repressor of p53, in cells with supernumerary centrosomes (Fava et al., 2017). Furthermore, inclusion of caspase-12 in inflammatory caspases has also been questioned as well as the engagement in ER-stress induced apoptosis (Lamkanfi et al., 2004).

**TABLE 1:** Classical categorisation of caspases in mice as in Shalini et al. (2015). Detailed information of functions and molecular signalling and general phenotype of caspase-deficient mice for each caspase is given by individual studies listed in the table.

Categorisation of caspases in mice			
	Caspase monomer	Caspase reviewed in	Caspase-deficient mice
Apoptotic initiators			
Caspase-2	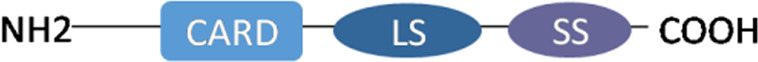	Brown-Suedel and Bouchier-Hayes (2020)	Bergeron et al. (1998), Zhang et al. (2007)
Caspase-8	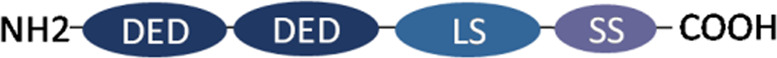	Mandal et al. (2020)	Varfolomeev et al. (1998)
Caspase-9	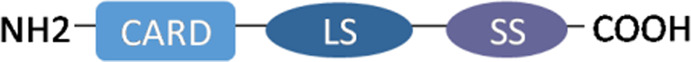	Li et al. (2017), Avrutsky and Troy (2021)	Kuida et al. (1998)
Apoptotic executors			
Caspase-3	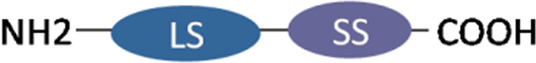	Asadi et al. (2022), Eskandari and Eaves (2022)	Leonard et al. (2002), Lakhani et al. (2006)
Caspase-6	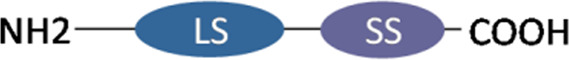	Wang et al. (2015)	Uribe et al. (2012)
Caspase-7	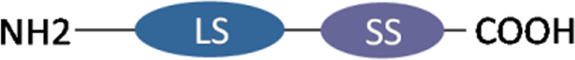	Lamkanfi and Kanneganti (2010)	Lakhani et al. (2006)
Inflammatory caspases			
Caspase-1	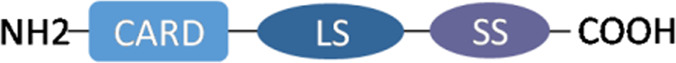	Sollberger et al. (2014)	Kuida et al. (1995)
Caspase-11	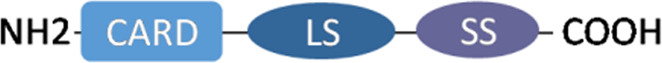	Agnew et al. (2021)	Wang et al. (1998b)
Caspase-12	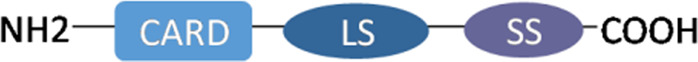	Dadley-Moore (2004)	Nakagawa et al. (2000), Skeldon et al. (2016)
Others			
Caspase-14	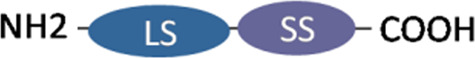	Denecker et al. (2008)	Denecker et al. (2007), Hoste et al. (2011)

CARD, caspase recruitment domain; DED, death effector domain; DD, death domain; LS, large subunit; SS, small subunit.

Caspases are expressed as zymogenic monomers, except for pro-caspase-9, which is a zymogen with a basal activity that is increased when activated (Stennicke et al., 1999). A monomer contains pro-domain, large and small subunits (Table 1). Long pro-domains are characteristic for initiator and inflammatory caspases, whereas executors only have the short one (Shalini et al., 2015) that affects their activation process (Ramirez and Salvesen, 2018). Long pro-domains of initiators carry either two death effector domains (DED) or caspase-activation recruitment domain (CARD) that promote recruitment and activation of these caspases in multiprotein complexes. The process of dimerization-induced autoactivation resides in the excision of the linker regions separating the pro-domain from the large and small catalytic subunits (Lamkanfi et al., 2002). In contrast, executioner caspases lack an extended pro-domain and require cleavage by initiator caspases to reach the activated state (Ramirez and Salvesen, 2018). Since inappropriate activation of caspases may be lethal, caspase activity regulation is ensured by a variety of cellular factors. This applies for posttranslational including phosphorylation, nitrosylation or ubiquitination, modifications that mostly result in decrease of caspase activation/activity. Phosphorylation is mediated by series of caspase specific kinases working at numerous sites (Parrish et al., 2013).

**FIGURE 1:**
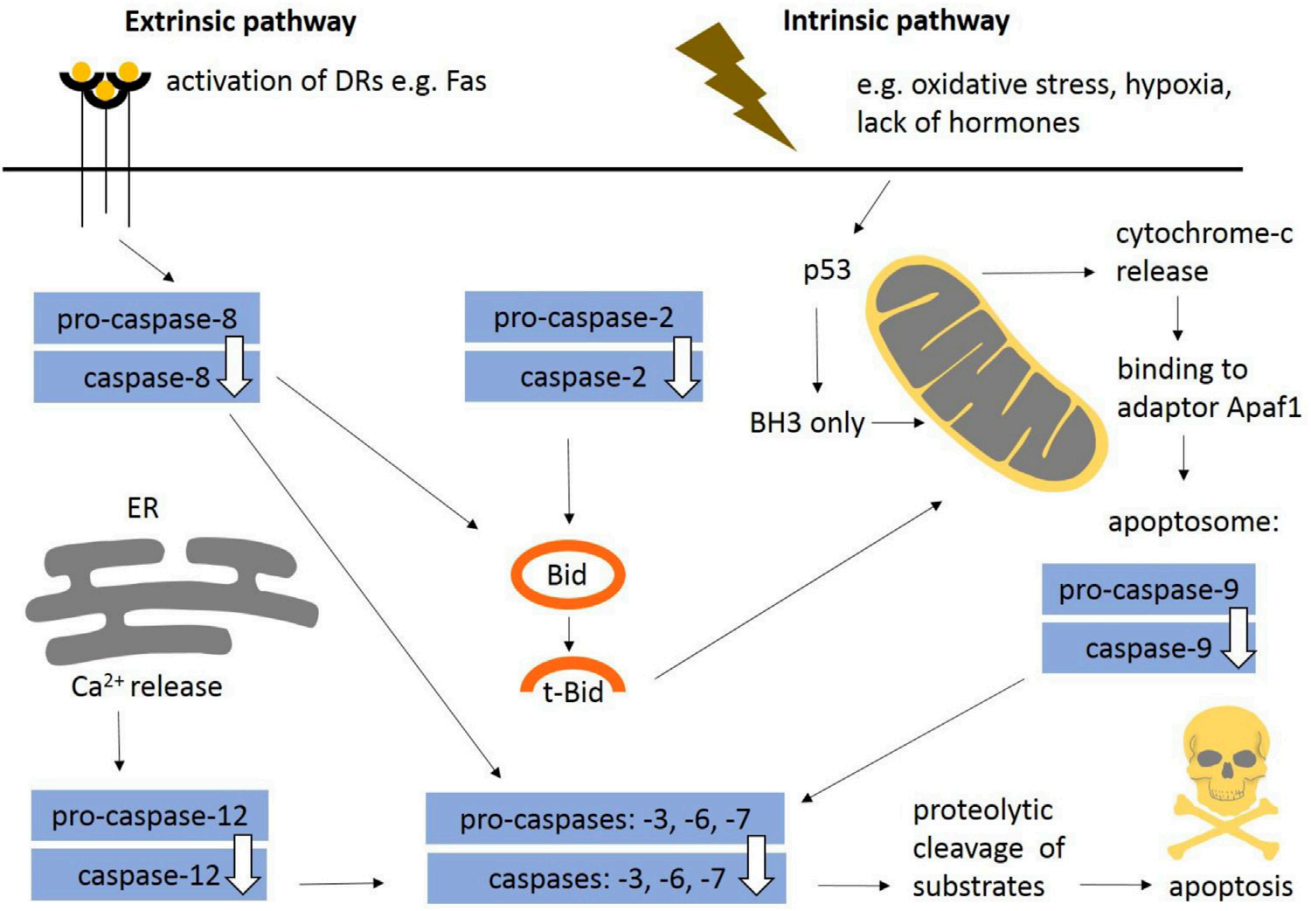
General schema of apoptotic engagement of caspases. The extrinsic pathway is induced *via* death receptors (e.g., Fas). Stimulation of death receptors results in activation of initiator caspase-8. The intrinsic pathway is usually initiated in a cell-autonomous manner (e.g., DNA damage, accumulation of unfolded/misfolded proteins, lack of pro survival factors such as cytokines). Internal signals regulate mitochondrial outer membrane permeabilisation and formation of apoptosome leading to activation of initiator caspase-9. Caspase-12 contributes to Ca^2+^ dependent apoptosis. Caspase-2 belongs to initiators. Both pathways aim to activate caspase-3, the central executor caspase, or other executors caspase-6, -7. The extrinsic and intrinsic pathways are often interconnected (e.g., Bid/tBid). Apoptosis is modulated by pro-survival and pro-apoptotic signals (BH3-only proteins). DRs, death receptors; ER, endoplasmic reticulum.

In general, apoptosis may be initiated by two distinct molecular pathways: extrinsic and intrinsic (Figure 1). The extrinsic pathway is triggered *via* death receptors (DRs) including a classical ligand-receptor interaction, such as Fas-FasL. The intrinsic pathway, also known as the mitochondrial apoptotic pathway, is usually initiated in a cell-autonomous manner, e.g., DNA damage, accumulation of unfolded/misfolded proteins, lack of pro-survival factors such as cytokines, hormones and growth factors, hypoxia, release of Ca^2+^, reactive oxygen species (Elmore, 2007; D’Arcy, 2019). Findings obtained from caspase-deficient animals showed that the intrinsic apoptotic pathway is essential for mammalian development (Voss and Strasser, 2020). The extrinsic pathway may also participate in morphogenesis (Svandova et al., 2017) and both mechanisms are often interconnected (Green and Llambi, 2015). Apoptotic pathways are modulated by pro-survival and pro-apoptotic members of the Bcl-2 protein family. The family consists of five pro-survival members—Bcl-2, Bcl-B, Bcl-xl, Bcl-w, Mcl-1, and A1/BFL1—and two pro-apoptotic subgroups. The pro-apoptotic BH3-only proteins (Bim, Puma, Bid, Bik, Bad, Bmf, Noxa, and Hrk) are critical for the initiation of apoptosis signalling *via* regulation of Bak and Bax activation (Ren et al., 2010), whereas Bax/Bak involved in mitochondrial outer membrane permeabilization (MOMP) are essential for the effector phase of apoptosis (Kale et al., 2018). Bok is a non-canonical pro-apoptotic protein controlled at the level of protein stability by components of the endoplasmic reticulum–associated degradation pathway (Carpio et al., 2015). Bok has not been directly proven to be a pro-apoptotic effector (Echeverry et al., 2013) and it is still unclear if Bok is able to perform MOMP on its own.

## Caspases: Killing or life-giving?

**FIGURE 2:**
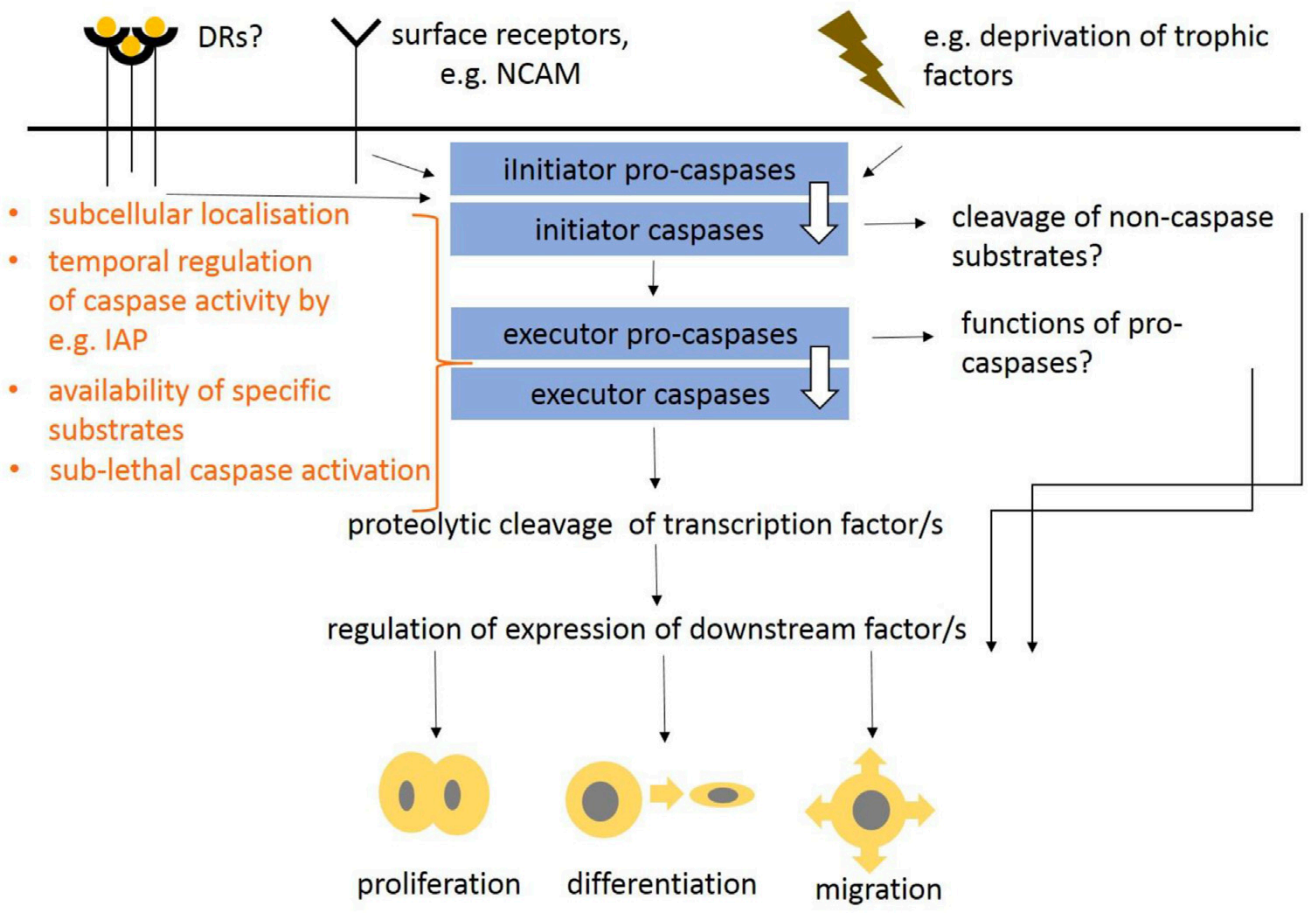
Putative schema suggesting non-lethal functions of caspases in intracellular signaling as proposed in Miura et al. (2004), Fujita et al. (2008), and Cusack et al. (2013). Non-apoptotic functions of caspases may be induced *via* death receptors, surface receptors (e.g., NCAM) or internal events, such as deprivation of trophic factors. This results in activation of various initiator caspases and potentially also executor caspases. The mechanisms regulating lethal vs. non-lethal events may reside in caspase subcellular localisation, availability of different substrates, temporal caspase activation or sub-lethal caspase activation. Functions of pro-caspases and proteolytic cleavage of non-caspase substrates is questionable. These regulations may result in proliferation, migration and differentiation of cells.

Caspases, that were first ascribed a role in apoptosis and inflammation (Poreba et al., 2013; Julien and Wells, 2017; D’Arcy, 2019; Van Opdenbosch and Lamkanfi, 2019; Kesavardhana et al., 2020), are now considered as multifunctional enzymes integrating lethal and non-lethal functions (Shalini et al., 2015). The most significant developmental defects come from insufficient or increased apoptosis that results in abnormal size of cell populations. In the head, the apoptotic effects are associated with the development of the nervous system, especially the brain, where insufficient apoptosis leads to increased populations of neurons leading to severe abnormalities (Kuida et al., 1996; Kuida et al., 1998; Cecconi et al., 1998; Hakem et al., 1998; Yoshida et al., 1998). Apoptotic caspase machinery including both, extrinsic and intrinsic pathways was previously defined including caspase interaction partners, regulators or down-stream molecules (as suggested above and overviewed in Figure 1). In contrast to apoptotic functions of caspases, the non-lethal ones are poorly understood but some possibilities have been suggested (Figure 2). The mechanisms that finally determinate lethal vs. non-lethal outcomes are still unknown but several theories have been proposed. The subcellular localisation of caspases was speculated as a promising solution (Prokhorova et al., 2018). The apoptotic breakdown of the nucleus is regulated by central caspase-3 (Walsh et al., 2008) which requires transport of caspase-3 into the nucleus (Kamada et al., 2005). Indeed, nuclear localisation of activated caspase-3 was identified in cells under apoptotic treatment (Prokhorova et al., 2018). By contrast, in non-apoptotic events, caspase activation is thought to be locally regulated in subcellular compartments, which results in availability of specific substrates (Nakajima and Kuranaga, 2017). Cytoplasmic localisation of caspase-3 was identified in cytoplasm of intact cells (Svandova et al., 2018). Sub-lethal caspase activation may be alternative way of non-apoptotic engagement (Basu et al., 2012). This may be connected with a phenomenon termed “minority MOMP” a process in which only a fraction of a cell mitochondria undergo permeabilisation. MOMP was initially identified as the mechanism leading to rapid caspase activation and apoptosis (Tait and Green, 2010). Minority MOMP leads to limited caspase activation, which is insufficient to trigger cell death. Instead, this caspase activity leads to DNA damage that, in turn, promotes genomic instability (Ichim et al., 2015). To prevent lethal caspase impact, caspase activity may be compensated by anti-apoptotic proteins (Huesmann and Clayton, 2006; Grabow et al., 2018) ensuring temporal control of caspase activity (Nakajima and Kuranaga, 2017). This is mediated by inhibitor of apoptosis proteins (IAPs). The temporal activation may be regulated by E3-ubiquitin ligase activity of IAPs promoting the degradation of caspases by the ubiquitin-proteasome system (Berthelet and Dubrez, 2013). Other mechanisms of caspase activity include post-translational modifications of caspases, or interaction with members of Bcl-2 protein family.

The non-lethal functions apply for various cell types and involve diverse processes (Lamkanfi et al., 2007; Connolly et al., 2014; Shalini et al., 2015) such as differentiation of skeletal myoblasts or osteoblasts, secretion and mineralisation of the enamel (Fernando et al., 2002; Miura et al., 2004; Matalova et al., 2013; Tisch et al., 2019). Abnormal cell differentiation observed in caspase-deficient mice was associated with abnormal gene expression. Based on the knowledge from caspase-deficient mice and inhibition experiments, caspases were shown to regulate the expression of genes engaged in various different pathways, such as Runx2 (Miura et al., 2004), Alpl, Bglap, Phex (Kratochvílová et al., 2020), Smad1, Msx1 (Svandova et al., 2014).

Caspases also regulate the course of the cell cycle and proliferation. This is mediated by proteolytic degradation of cell-cycle components, such as p21, p27, CDK11, or p105Rb (Connolly et al., 2020). Migration belongs to other crucial non-lethal function of caspases, where caspases cleave cytoskeleton components such as actin (Mashima et al., 1997).

## Substrates to be ruined

Almost 400 caspase substrates are known (Lüthi and Martin, 2007), however, an exhaustive view of the specific roles of each member of the caspase family is lacking (Nguyen et al., 2021). It is very likely that many more caspase substrates remain to be identified but identification is hindered by the difficulties of distinguishing functionally important caspases substrates from proteolytic noise. The interpretation of the observations is further complicated by *in vitro* conditions, where recombinant caspases are used in high concentrations, and therefore the outcomes may not be the same as *in vivo*. Caspases are not equal in terms of their proteolytic ability. Initiator caspases appear to cleave few substrates apart from their own precursors and other caspases downstream while effector caspases have a broader spectrum of targets. Also, within the individual groups there are differences in caspase substrate preferences. Among executors, caspase-3 seems to be more promiscuous compared to caspase-7 in terms of apoptotic machinery (Porter and Jänicke, 1999; Slee et al., 2001).

Caspase substrates are categorised as several groups, mostly in the context of apoptotic machinery (Fischer et al., 2003; Lüthi and Martin, 2007) but the spectrum has gradually expanded. Caspase substrates include proteins associated with apoptosis, cell adhesion, regulation of the nuclear structure, formation of the cytoskeleton, physiology of endoplasmic reticulum and Golgi apparatus, cell cycle, DNA synthesis, cleavage and repair, etc. (Fischer et al., 2003). Fewer caspase substrates are identified in non-apoptotic processes, this applies to Sema7a during sensory neuron maturation (Ohsawa et al., 2010), Mst1/STK4 (Fernando et al., 2002), Pax7 for the differentiation of skeletal muscles (Dick et al., 2015) or βcat for cardiac muscle cells (Abdul-Ghani et al., 2011).

## Apoptotic functions of caspases during development of the head

A normal rate of apoptosis is essential to fulfil morphogenetic functions. In mammals, apoptotic cells are apparent already in the morula (Fabian et al., 2007) and apoptosis later accompanies morphogenesis of various head structures (Pampfer and Donnay, 1999) (Table 2).

**TABLE 2:** Overview of caspase apoptotic functions during development of head.

Apoptotic engagement of caspases—summary		
Brain	Regulation of neural cells population by elimination of excessive neurons: **casp-3, -9 and Apaf-1, Bax**	Kuida et al. (1996), Cecconi et al. (1998), Hakem et al. (1998), Jung et al. (2008)
	Protection of neurons: **casp-2**	Bergeron et al. (1998)
	Neural tube formation: **casp-8**	Varfolomeev et al. (1998), Sakamaki et al. (2002)
	Development of motoneurons, sympathetic and sensory neurons: **Bcl-2**	Michaelidis et al. (1996)
Eye	Apoptosis in the inner nuclear layer of retina: **casp-3**	Zeiss et al. (2004)
	Lens transparency: **casp-3**	Ishizaki et al. (1998), Zandy et al. (2005)
Inner ear	Abnormal anatomy of inner ear: **casp-3**	Makishima et al. (2011)
	Decreased apoptosis in the inner ear epithelium: **casp-9, Apaf-1**	Cecconi et al. (2004)
Skull	Activation in apoptotic cells of mandible during prenatal development: **casp-3, -7 and -8**	Svandova et al. (2018)
Palatal shelves	Fusion of palatal shelves *ex vivo*: **General caspase activity**	Cuervo et al. (2002)
	Lack of adherence in palatal shelves: **Apaf-1**	Honarpour et al. (2000)
	Absence of MES disintegration: **Apaf-1**	Cecconi et al. (1998)
Teeth	Apoptosis of cells in primary enamel knot: **casp-3, -9 and, Apaf-1**	Setkova et al. (2007)
Skeletal muscles	Destruction of myonuclei and myofibers in aging tongue muscle: **casp-3**	Kletzien et al. (2018)
Skin	Skin homeostasis: **casp-8**	Kovalenko et al. (2009)
	Melanin synthesis: **Bcl-2**	Veis et al. (1993)
Salivary glands	Temporarily restricted regulation of duct size in salivary glands *ex vivo*: **General caspase activity**	Teshima et al. (2016b)

Apoptosis-related molecules are shown in bold font.

The most significant impact of insufficient apoptosis was observed during the formation of the nervous system. Apoptotic elimination of neurons is required to adjust their number. At the beginning of neural development, excessive numbers of neurons is generated including many with incorrect connections to their targets. Later, some are removed by caspase-mediated apoptosis (Volbracht et al., 2001). Caspase-3, -9 and, Apaf-1, were shown to be essential for this process. Mice lacking either of these factors showed severe malformations resulting from an excessive number of neurons (Kuida et al., 1996; Kuida et al., 1998; Cecconi et al., 1998; Hakem et al., 1998; Yoshida et al., 1998; Honarpour et al., 2000). Notably, caspase-3-deficient mice revealed inconsistent phenotypes related to the genetic background (Leonard et al., 2002). *Caspase-3* ^−/−^ 129X1/SvJ mice were severely affected by expansion of neural precursors and exencephaly (Kuida et al., 1996; Woo et al., 1998). However, caspase-3-deficient C57BL/6J mice showed minimal brain pathology (Leonard et al., 2002) suggesting compensatory caspase activation or expression/activation of other proteins depending on the specific genetic background (Zheng et al., 2000; Leonard et al., 2002). Caspase-3-deficient mice C57BL/6 displayed an increased activation of caspase-7 compared to 129X1/SvJ (Houde et al., 2004). The role of different genetic backgrounds was identified also in non-caspase knock-out studies and suggested to influence the phenotypic analysis (Montagutelli, 2000). Supplementary Table S1 summarises genetic backgrounds in mice described in this review. Notably, caspase-2 seemsto work in a different manner to caspase-3, -9 and Apaf-1. Caspase-2 deficiency caused a decreased number of facial neurons suggesting a protective function in neural development. Other abnormalities were not detected in the brain of caspase-2-deficient mice (Bergeron et al., 1998). Caspase-8-deficient mice displayed neural tube malformations. However, these alterations are probably secondary effects as a consequence of failure in caspase-8-mediated apoptosis allowing the survival of some cells committed to death (Varfolomeev et al., 1998; Sakamaki et al., 2002). Mice lacking inflammatory caspase-1, -11, -12 did not display any obvious neuronal phenotype (Kuida et al., 1995; Li et al., 1995; Wang et al., 1998b; Saleh et al., 2006; Kayagaki et al., 2011). Caspase-1 was only shown to decrease hippocampal neurogenesis with ageing (Gemma et al., 2007). Neither caspase-6 nor caspase-7 appear to be critical for the central nervous system development (Houde et al., 2004; Lakhani et al., 2006; Uribe et al., 2012). Regarding caspase regulation, ablation of the pro-apoptotic gene Bax, which appears to be critical for post-mitotic neuronal cell death (White et al., 1998), led to a selective reduction in the elimination of neurons (Jung et al., 2008). Bcl-2-deficient mice exhibited an important loss of motoneurons, sympathetic neurons, and sensory neurons during early postnatal life (Michaelidis et al., 1996). The importance of fine regulation of neural apoptosis was enhanced in *Mcl-1* ^*+/−*^ *Bcl-x* ^*+/−*^mice, where small but excessive increase in developmental apoptosis resulted in a high incidence of developmental abnormalities of head including hydrocephalus (Grabow et al., 2018).

Defective apoptosis impacts development of other neuroectoderm-derived structures including the retina. The development of the retina in vertebrates is accompanied by a physiological cell death (Vecino et al., 2004). The sequence of cell death in the retina probably recapitulates the sequence of maturation in the various layers and cell types, starting in the ganglion and proceeding across the inner and outer nuclear layer (Beazley et al., 1987). Caspase-9 was shown to be an initiator of the process (Laguna et al., 2008) and central executor caspase-3 follows in the machinery. In caspase-3-deficient mice (maintained on a mixed C57BL/6J-129sv background), apoptosis was retarded in the inner nuclear layer of the retina. The compensation implies a caspase-independent mechanism together with a caspase-dependent mechanism mediating cell death. The inhibition of one results in activation of the other (Zeiss et al., 2004). The caspase-independent mechanism may include apoptosis-inducing factors (Candé et al., 2002) or non-caspase proteases (Guicciardi et al., 2001).

During lens development, primary and secondary lens fibres lose their nuclei and organelles to form a transparent cytoplasm. In several aspects this process resembles apoptosis, since it includes TUNEL-positive degenerating nuclei stained, activated caspases, cleaved poly-(ADP-ribose) polymerase (PARP) etc. (Appleby and Modak, 1977; Bassnett and Mataic, 1997; Zandy et al., 2005). However, some parameters are different from what is observed during apoptosis incuding maintenance of phosphatidylserine within the inner leaflet of cytoplasmic membrane, and/or the elongation and preservation of cells (Bassnett and Beebe, 1992; Bassnett and Mataic, 1997; Ishizaki et al., 1998; Wride and Sanders, 1998; Dahm, 1999). Whether the denucleation of cells resulting in maturation of lens fibres is apoptotic or not remains unclear. Dahm (1999) or Sanders and Parker (2002) suggested that lens fibre cell denucleation is an apoptotic-like event lacking plasma membrane phenomena associated with apoptosis. This could be associated either with the relatively early permeability changes in the mitochondria and the consequent loss of activated caspase-9 or other mitochondrial proteins. Alternatively this may involve the failure of signalling molecules to migrate to the nuclei (Sanders and Parker, 2002). These authors used lens epithelial cultures to show that pharmacologic inhibition of caspases-1, -2, -4, -6, and -9 significantly reduced the incidence of nuclear degeneration, whereas inhibitors of caspases-3 and -8 did not.

The survival of lens fibres was suggested to be involved within large networks of gap junctions ensuring cellular communication. Caspase-3 was shown to be responsible for proteolytic cleavage of connexin, a gap junction protein, associated with lens development (Yin et al., 2001). Basu et al. (2012) described development of chick lens fibres as a process of differentiation, where low levels of caspase-3 activation is regulated by IGF-1R/NFκB signalling and caspase-3-deficient mice exhibited cataracts (Zandy et al., 2005). However, further investigation showed that neither caspase-3 nor other executors are required for the elimination of organelles from lens fibres (Zandy et al., 2005).

In addition to caspases, other apoptosis-related factors have been identified in the process of lens development. For instance, over-expression of Bcl-2 in the chick lens results in morphological defects, including disorganised lens fibres. In the equatorial region where cells begin to differentiate, pro-apoptotic Bcl-2 family members (Bax and Bcl-Xs) are expressed, which might be an initial signal for cell differentiation (Sanders and Parker, 2003). Surprisingly, over-expression of Bcl-2 was sufficient to induce cataracts, microphakia, vacuolisation, fibre cell disorganisation, and inhibition of fibre cell denucleation (Fromm and Overbeek, 1997).

Apoptosis was reported as a possible key event (although not the only one) in eyelid spacing (Mohamed et al., 2003). Caspase activity would be expected in the process however, no obvious alterations in eyelids have been described in mice lacking the central caspase-3 (Zeiss et al., 2004). Therefore, alternative mechanisms of cell death or alternative processes of morphogenesis are expected.

Among sensory organs, the inner ear is an example where a balanced rate of apoptosis is believed to be crucial for correct anatomy of vestibular system (Tafra et al., 2014). Caspase-3-deficient mice showed inconsistent phenotypes with prevalent decreased arc size of the anterior semicircular canal. Other severe malformations included truncation or aplasia of the anterior semicircular canal (Makishima et al., 2011). Furthermore, absence of Apaf-1 led to a dramatic decrease in apoptosis in the epithelium of the inner ear, severe morphogenetic defects and a significant size reduction of the membranous labyrinth. Caspase-9-deficient mice suffered from similar defects supporting the importance of the Apaf-1-caspase-9-caspase-3 pathway. The aforementioned phenotype is speculated to come from the reduction in the number of apoptotic cells and thus of the passive release of functional factors from the dying cells into the local environment (Cecconi et al., 2004). Alternatively, persisting unwanted cells may release signals incompatible with normal development. Parker et al. (2010) suggested apoptotic function of caspase-3 also in auditory part of the inner ear. Loss of hair cells and spiral ganglia was reported in caspase-3-deficient mice (Takahashi et al., 2001). The phenotype of caspase-3-deficient mice included hyperplasia of supporting cells of organ of Corti. That probably results from abnormal cell elimination. Hair cells degeneration was, however, associated with non-apoptotic events (Morishita et al., 2001).

Apoptosis was observed to regulate the final shape and size of skeletal muscles. In addition apoptosis was associated with atrophy of skeletal muscles during ageing (Sandri and Carraro, 1999; Schwartz, 2018). Increasing rate of apoptosis was identified in ageing of extrinsic tongue muscle and caspase-3 may be involved in this process (Kletzien et al., 2018).

Development of the skull includes the differentiation of connective tissues such as cartilage, bone or associated dental tissues. Apoptotic removal of bone cells keeps a balance between the number of bone-forming osteoblasts, bone resorbing osteoclasts, and mechanical sensors known as osteocytes, which is crucial for bone formation and physiology. Inappropriate apoptosis in bone may be responsible for pathologies such as osteoporosis or rheumatoid arthritis (Hughes and Boyce, 1997; Mollazadeh et al., 2015; Ru and Wang, 2020). Caspase-3, -7, and -8 activation was associated with apoptosis in mandibular bone cells (Svandova et al., 2018). Additionally, caspase-2 was reported to be involved in maintaining bone homeostasis by modulating the levels of reactive oxygen species in osteoclast apoptosis during ageing (Sharma et al., 2014).

Formation of the secondary palate requires direct contact and fusion of the palatal shelves in the temporal structure medial epithelial seam (MES). Incomplete fusion of the palatal shelves results in palatal clefts, the most common congenital craniofacial deformity. Apoptosis was shown to contribute to the disintegration of the MES. However, the role of caspases in palatal development remains disputable. Organ cultures demonstrated that the application of general caspase inhibitor results in persistence of the MES and unfused palate shelves (Cuervo et al., 2002). Furthermore, Apaf-1-deficient mice displayed a lack of adherence in palatal shelves (Honarpour et al., 2000) or absence of MES disintegration (Cecconi et al., 1998). Finally, treatment with a blocking FasL antibody in organ culture prevented palatal fusion and inhibited the expression of caspase-8 and -3 (Huang et al., 2011). By contrast, Jin and Ding (2006) did not confirm a necessity for Apaf-1-dependent apoptosis for normal palatal development as demonstrated in Apaf-1-deficient mice. The discrepancies might result from different genetic backgrounds. Notably, caspase-3 or caspase-9-deficient mice did not show cleft palate (Kuida et al., 1996; Kuida et al., 1998; Woo et al., 1998), which again reduces the importance of apoptosis-related molecules in palate development. Therefore, some alternative mechanisms such as cell trans-differentiation, migration or some other still unknown mechanism are expected to take a part in the process (Jin and Ding, 2006).

Caspase-7 is activated during apoptosis that takes place in the primary enamel knot (PEK), a signalling center involved in tooth morphogenesis. Despite its activation, caspase-7 does not seem to be crucial for the process (Matalova et al., 2012). In contrast, deficiency of caspase-3, caspase-9, and Apaf-1, respectively, resulted in the suppression of apoptotic elimination. Surprisingly, despite these markers being abundantly present in PEK, their deficiency does not affect the normal formation of dental structures (Matalova et al., 2006; Setkova et al., 2007). An impact of caspase-3 deficiency on epithelial formation and enamel structure was investigated using different genetic backgrounds. At prenatal development, the location of the first molar tooth germ was found shifted posteriorly in the upper jaw in *Caspase-3* ^*−/−*^/129X1/SvJ mice, which might be related to the enlarged brain in these mice. *Caspase-3* ^*−/−*^on the B57BL/6 background altered morphology of the first molar in both upper and lower jaws, with the original PEK epithelium appearing disorganised (Matalova et al., 2006). The variability of phenotypes may be related to the specific pattern of redundant caspase activation. Despite the prenatal differences of tooth organisation, no major alterations in adult molars in both strains were described (Leonard et al., 2002).

Lumen formation is crucial for function of salivary glands. In mice, apoptosis was first apparent in epithelial stalks together with cleaved caspase-3 immunodetection. Application of general caspase inhibitors to *ex vivo* cultures resulted in wider ducts, and a defect in lumen formation, compared with controls. In contrast, no such defect in lumen formation was observed at later stages pointing to a temporarily-restricted action of caspases on cell elimination (Teshima et al., 2016b). Caspase-7 was evident earlier during development, while caspase-6 was mainly concentrated within more developed ducts. Therefore, their functions are rather complementary. The prevailing expression of Bax and Bak to Bad and Bid in developing human salivary glands again strengthens the importance of intrinsic apoptotic pathway in developmental events (Teshima et al., 2016a).

Apoptotic and inflammatory caspases were identified in skin (Takahashi et al., 1998) and the hair buds. Prenatal development of the hair buds was not associated with significant apoptosis (Vesela et al., 2015). However, adult hair follicles undergo periodic hair cycling (Cotsarelis, 2006). Soma et al. (1998) observed apoptotic cells in human inner root sheaf and identified expression of caspase-1, -3, -4 (analogue of caspase-11) and -7 during the hair growth (anagen). In mice, at the stage of growth arrest (catagen) apoptosis has been shown to occur in the inner root sheath and the lower part of the follicle, apoptotic cells were associated with activation of caspases-3, -7, -12 (Vesela et al., 2015; Veselá and Matalová, 2015). Caspase-9 was shown to regulate apoptosis in hair follicle stem cells (SCs). Caspase-9-deficient hair follicle SCs displayed high levels of caspase-3. Surprisingly, caspase-3 activation was not sufficient for SCs elimination. SCs of hair follicle were retained in an apoptotic-engaged state, during which they released mitogenic Wnt3. Consequently, caspase-9-deficient mice accelerated wound repair and hair follicle regeneration (Ankawa et al., 2021). The role of caspase-3 in the context of SCs differentiation was described also in embryonic stem cells (Fujita et al., 2008). Another factor regulating hair follicle turn-over is Bcl2 (Stenn et al., 1994; Sotiropoulou et al., 2010). Recently, it was shown to regulate homeostasis of hair follicle stem cells (Geueke et al., 2021).

## Non-apoptotic functions of caspases during development of the head

Caspases have recently been found to be involved in processes such as cell proliferation, adhesion, differentiation or migration. Importantly, the same systems, where apoptotic engagement of caspases was identified, are associated with their non-apoptotic activation (Table 3).

**TABLE 3:** Overview of caspase non-lethal functions during development of head.

Non-lethal engagement of caspases—summary		
Brain	Regulation of neural differentiation: **casp-2,-3,-9**	Fernando et al. (2005), Aranha et al. (2009), Pistritto et al. (2012)
	Reorganisation of cells during neural tube closure *ex vivo*: **General caspase activity**	Shinotsuka et al. (2018)
	Axon branching: **casp-3, -9**	Campbell and Okamoto (2013), Katow et al. (2017)
	Axon pruning: **casp-3,-6, -9**	Cusack et al. (2013)
	Axon guidance: **casp-9, Apaf-1**	Ohsawa et al. (2010)
Eye	Vascularisation of retina: **casp-8**	Tisch et al. (2019)
	Proper thickness of retina: **Apaf-1**	Honarpour et al. (2000)
	Proper development of retina and lens: **Bcl-2 superfamily**	Mosinger Ogilvie et al. (1998), Sanders and Parker (2003)
Inner ear	Development of spinal ganglion and hair cells: **casp-3**	Morishita et al. (2001), Takahashi et al. (2001)
Skull	Ossification of the skull: **casp-3**	Miura et al. (2004)
	Bone volume of mandible: **casp-7, Fas/FasL**	Svandova et al. (2014), Svandova et al. (2019)
Teeth	Mineralization of enamel: **casp-7**	Matalova et al. (2012), Matalova et al. (2013)
Skeletal muscles	Differentiation of skeletal muscles: **casp-3, -8**	Fernando et al., 2002
Skin	Terminal differentiation of keratinocytes: **casp-14**	Lippens et al. (2000)
	Regulation of mast cells population: **casp-7**	Vesela et al., 2015
Blood vessels	Proliferation, sprouting and migration of endothelial cells: **casp-8**	Varfolomeev et al. (1998)

Apoptosis-related molecules are shown in bold font.

Multiple non-apoptotic caspase functions were identified in the nervous system (Nguyen et al., 2021). The importance of non-apoptotic functions of caspase-3 was shown in the mouse primary neuronal SCs, where a deficiency of caspase-3 resulted in abnormal signals for cytoskeletal remodelling (Fernando et al., 2005). Additionally, the presence of *β*-III tubulin-positive and GFAP-positive cells was reduced in mouse neural stem cells when caspase activity was inhibited (Aranha et al., 2009). In postnatal rat cerebellum, caspase-3-positive cells were localised in the external granule cell layer and did not correlate with apoptosis. In this model, caspase-3 was suggested to be engaged in the reorganisation of components of the cytoskeleton such as actin (Mashima et al., 1997), fodrin (Martin et al., 1995; Greidinger et al., 1996), and spectrin (Wang et al., 1998a). Caspase-3 was also suggested to accompany neural mitosis or differentiation (Oomman et al., 2004). Furthermore, caspase-3 expression was not limited to neural cells but was also detected in differentiating Bergmann glia of the cerebellum (Oomman et al., 2006). In chick, non-apoptotic engagement of caspase-3 was identified in the auditory brainstem, where caspase-3 substrates were found to be expressed in axons (Weghorst et al., 2020). In addition to caspase-3, other caspases, such as caspase-2 and -9 are suspected to participate in neural differentiation (Pistritto et al., 2012).

Caspases have been shown to be important for neural tube closure (NTC). During this process the boundaries between the non-neural ectoderm and the neuroepithelial layer are provided by cells undergoing apoptosis suggesting a participation of caspases in the process (Geelen and Langman, 1977; Weil et al., 1997). Caspase-3, -9 and Apaf-1-deficient mice displayed NTC defects in the midbrain and/or the hindbrain. Surprisingly, the closure proceeds normally in other parts (Hakem et al., 1998; Kuida et al., 1998). The phenotype, therefore, may not be a direct consequence of the suppression of apoptosis but could be due to the abnormal persistence of certain signal-secreting cells. Alternatively, caspases may also contribute to the closure in a non-apoptotic manner. Experiments in early mouse embryos showed that a general caspase inhibitor prevents cell migration of non-neural ectodermal cells and normal NTC (Shinotsuka et al., 2018).

Finally, caspases were identified in the regulation of axonal development including axon branching, pruning (axon retraction), guiding (axon navigation) and the formation of synapses. Most of these studies were however carried out in non-mammalian animal models. Activation of caspase-3 has been transiently observed in axons, particularly at their branching points (Campbell and Okamoto, 2013; Katow et al., 2017). The mechanisms by which caspase-3 regulates growth cone formation and axon branching are still elusive. Actin polymerization is necessary to drive advancement of neuronal growth cones (Dent et al., 2011) preceding synaptogenic events. Since caspase-3 recognises cytoskeletal compartments such as actin (Mashima et al., 1997) it may be a possible mechanism. Axon pruning is a process that eliminates collateral extensions or small terminus arborisation with improper connectivity at the axon terminus. Selective pruning of axons is critical for plasticity in the adult nervous system (Low and Cheng, 2006). Whereas neuronal cell death occurs early during neural development, axon pruning continues to be selectively removed at least through adolescence in humans (Sakai, 2020). Axon-selective degeneration requires caspase-9 (Cusack et al., 2013) and caspase-3 (Simon et al., 2012). Suppression of caspase-3 activation in Bax/Bak double knock-outs led to a less tailored postnatal network of neuron branches in the spinal cord and impaired the development of skilled movements in adult mice (Gu et al., 2017). Notably, axon pruning does not require Apaf-1 (Cusack et al., 2013) but is dependent on caspase-6 (Nikolaev et al., 2009; Simon et al., 2012; Cusack et al., 2013). In this context, caspase-6 is not essential for neuronal death (Cusack et al., 2013). Caspase signalling is suspected to regulate axon guidance and differentiation. As such Apaf-1/caspase-9 signaling caused the cleavage of Sema7A, crucial for proper axon projection, which results in a decreased level of its active form in olfactory sensory neurons. Consequently, mice deficient for Apaf-1 or caspase-9 exhibit misrouted axons (Ohsawa et al., 2010). Caspases were further investigated in the context of the processes of learning and memory. Caspase-3 plays an important role, as shown in zebrafinch (Huesmann and Clayton, 2006). Caspase-3 is also involved in synaptic plasticity (D’Amelio et al., 2012), notably in long-term depression (LTD), a process during which the efficiency of synaptic transmission is reduced for hours. As a counterpart to long-term potentiation (LTP), it is also important for adapting neural networks to physiological activity requirements.

Apaf-1-deficient mice displayed retinal thickening (Honarpour et al., 2000). Caspase-3–deficient animals suffer from marginal microphthalmia, peripapillary retinal dysplasia and delayed regression of vitreal vasculature. It is questionable whether some of these abnormalities might result from non-apoptotic mechanisms (Zeiss et al., 2004). Investigation of rat lens proposes a putative role of caspase-3 in turnover of lens proteins caused by incident light (Talebizadeh et al., 2015). The development of retina is also impacted by genetic alterations of the Bcl-2 superfamily and anti-apoptotic proteins (e.g., Mosinger Ogilvie et al., 1998; Strettoi and Volpini, 2002).

In the auditory region of the inner ear, non-apoptotic functions of caspases have also been identified. *Caspase-3* ^*−/−*^ mice were shown to exhibit a marked degeneration of the spiral ganglion neurons and a loss of inner and outer hair cells in the cochlea with ageing. The degenerated neurons did not exhibit characteristics of apoptosis. This observation suggests a role of caspase-3 in the survival of ganglia and hair cells (Morishita et al., 2001).

Caspase-3 is indispensable for the transition of skeletal myoblasts into myotubes and expression of muscle-specific proteins. *In vitro* analysis showed that skeletal muscle differentiation is accompanied by a significant increase in caspase-3 activity. Furthermore, the increasing trend (not as large as for caspase-3) was evident also for caspase-8 (Fernando et al., 2002). Caspase-3 was further associated with catabolic degradation of muscle proteins (Du et al., 2004). *In vitro* investigation of C2C12 myoblasts showed that caspase-9 is required for caspase-3 activation and cell fusion. Reduction of caspase-9 levels prevented caspase-3 activation. By contrast, the processing of other apoptotic initiator caspases was not detected (Murray et al., 2008). General caspase activity was demonstrated in the regulation of muscular regeneration (Moresi et al., 2009). Later studies identified that caspase-3 limits satellite cell self-renewal *via* inactivation of Pax7 (Dick et al., 2015). Satellite cells are responsible for the developmental growth and the regeneration of muscles.

Deficiency of caspase-3 resulted in delayed skeletal development of bones of the skull including the ossification defects of calvarial bones. However, it is possible that the enlarged brain in *caspase-3* ^−/−^ may contribute to the phenotype. Nevertheless, the delayed ossification of the skull may also result from a significant decrease in expression of Runx2/Cbfa1 in *caspase-3* ^*−/−*^ and *caspase-3* ^*+/–*^ detected in pre-osteoblasts derived from mouse calvariae (Miura et al., 2004).

In contrast to caspase-3-deficient mice, caspase-7 deficiency did not result in any observable change in the size of the skull when compared with the normal littermates. Analysis of *caspase-7* ^*−/−*^ adult mandibular bone revealed that the bone mineral density (BMD) was comparable to that of wild-type animals. However, in *caspase-7* ^*−/−*^ mandibles, the bone volume was significantly decreased compared with wild types, which correlates with significant decrease in Msx1 and Smad1 expression, both involved in bone formation (Svandova et al., 2014). Smad1 is one of the key players in the Bone morphogenetic protein (BMP) pathway and induces bone formation (Nohe et al., 2004), which might explain the decreased bone volume in young adult mice. The potential of caspases to regulate osteogenic expression was also studied in calvarial MC3T3-E1 cells. A sequence of *in vitro* experiments with caspase inhibitors showed downregulation of osteocalcin (Ocn) and Phex. The inhibition of individual caspases indicated that caspase-8 is a major contributor to the decrease in Ocn and Phex expression. Caspase-2 and-6 inhibition decreased expression of Ocn and caspase-6 inhibition decreased Phex expression (Kratochvílová et al., 2020). *In vitro* modulation of osteoblast differentiation *via* caspase-2, -3, -8 was observed (Mogi and Togari, 2003). Furthermore, caspase-12 was shown to regulate the expression of alkaline phosphatase, osteocalcin and Phex *in vitro* and was activated in osteoblasts of mandibular bone *in vivo*. The increasing expression of caspase-12 during development of mandibular bone, when the original mesenchymal condensation turns into vascularised bone, suggests a role in osteoblast differentiation (Vesela et al., 2020). Impacted bone formation was recorded also in the case of caspase upstream factors such as FasL, a regulator of the extrinsic apoptotic pathway. FasL-deficient mice displayed abnormal expression of Mmp2 and Sost in prenatal mandible, both factors are important regulators of bone formation. Furthermore, FasL-deficient mandibles showed age-dependent phenotype, when 6-day old mice had decreased and 24-day old mice increased bone volumes (Svandova et al., 2019). Non-apoptotic effects of FasL was shown also in osteocyte lineage IDG-SW3, where stimulation of these cells resulted in abnormal expression of osteogenic genes with the most downregulated gene being sclerostin (Kratochvilova et al., 2021).

Caspase activation during tooth development was further identified in non-apoptotic odontoblasts (in crown and root) and ameloblasts. Caspase-3-deficient first molars did not show any significant alterations when compared with controls (Matalova et al., 2006; Setkova et al., 2007). This contrasts with the situation observed in the *caspase-7^−/−^* mice where incisors displayed delayed mineralisation or hypomineralisation of the enamel. Notably, caspase-7 has a different localisation in the epithelial cells on the lingual side of rodent incisor where enamel is not secreted (caspase-7 negative) and the labial side of continuously renewing ameloblasts (caspase-7 positive). The activation of caspase-7 in the cervical loop suggested a possible role of caspase-7 in fate of dental cells engaged in the formation of extracellular matrices and mineralisation (Matalova et al., 2013). Caspase-12, also investigated during odontogenesis, was activated in differentiating ameloblasts and odontoblasts, although its exact function is not yet known (Vesela et al., 2020).

During skin development, caspase-14, which is believed to have functions unrelated to apoptosis (Hu et al., 1998), was shown to be crucial for the terminal differentiation of keratinocytes (Lippens et al., 2000). Caspase-14 is expressed in the differentiating suprabasal keratinocytes (Lippens et al., 2004; Hoste et al., 2011). However, based on its expression, it probably participates in skin barrier formation (Hu et al., 1998). One of the essential functions of caspase-14 is the processing of profilaggrin, a protein essential for moisturisation of the stratum corneum (Hoste et al., 2011) resulting in increased trans-epidemal water loss and sensitivity to UVB radiation (Denecker et al., 2007). Caspase-7 was suggested to regulate the number of mast cells localised in the dermis (Vesela et al., 2015). The non-apoptotic pathway might include a proteolytic cleavage of IL-33, an activator of mast cells (Sabatino et al., 2012; Saluja et al., 2014) and also a substrate of caspase-7 (Lüthi et al., 2009).

Caspases-1, -12, -7, -14 were present in developing hair germs in non-apoptotic cells and therefore non-apoptotic functions have been suggested (Vesela et al., 2015). Caspase-14 was diffusely present in cornifying cells of the outer root sheath, in the companion layer, and in the inner root sheath (Alibardi et al., 2005). Caspase-1 was shown to impact cell proliferation in different niches of the skin (Lee et al., 2015). Additionally, hair growth in caspase-2-deficient mice was impaired (Zhang et al., 2007).

Caspase-8-deficient mice showed abnormal formation of blood vessels (Varfolomeev et al., 1998). Loss of caspase-8 in endothelial cells results in decreased proliferation, sprouting and network formation. Loss of caspase-8 caused hyperactivation of p38 MAPK within the receptor-interacting serine/threonine protein kinase 3 (RIPK3) pathway and destabilisation of endothelial cadherin (VE-cadherin) (Tisch et al., 2019). Caspases have been shown to be involved in degradation of extracellular matrix (Cowan et al., 2005) which may be applied also in remodelling of blood vessels. Such an activation is expected for caspase-3 (Cohen, 1997), -2, and -7 (Cowan et al., 2005).

## Concordance between knockout animals

Regarding the apoptotic processes, there are several similarities in mice lacking apoptosis-related factors. As indicated above, the trio of knockout mice caspase-3, -9 and Apaf-1 was shown to be essential for several events; elimination of the excessive number of neurons (Kuida et al., 1996; Kuida et al., 1998; Cecconi et al., 1998; Hakem et al., 1998; Yoshida et al., 1998; Honarpour et al., 2000), apoptosis in the epithelium of the inner ear (Cecconi et al., 2004), removal of PEK cells (Matalova et al., 2006; Setkova et al., 2007). Notably, for non-apoptotic events similar group of molecules was not identified. This may suggest more variable pathways for non-apoptotic processes in contrast to apoptotic pathways. We may also speculate about less/more conserved mechanisms when compared these two groups.

## Evolutionary conserved functions of caspases in development of head structures

Caspases and caspase-like proteins are highly evolutionary conserved enzymes identified in vertebrates, insects, nematodes, or yeast. Their evolutionary conserved functions are not surprising in development of the head. This applies to the nervous system, where (Hakem et al., 1998; Kuida et al., 1998), caspase-9 was shown regulate size of neuron population in chick (Tafreshi et al., 2006). Caspase-9 was identified also in the developing nervous system in zebrafish (Spead et al., 2018). Caspase activity (either drICE or dcp-1) was also identified to be essential for neuronal death in *Drosophila* (Akagawa et al., 2015). Caspases were identified to participate in neural tube closure in both mice (Hakem et al., 1998; Kuida et al., 1998) and chick embryos (Weil et al., 1997). Furthermore, there is a potential link between mutations in caspase-3, -9 and Apaf-1 and neural tube closure defects in humans (Nguyen et al., 2021).

Caspase engagement in development of the eye was detected in vertebrates (Beazley et al., 1987) but also in the compound eye of *Drosophila*, where apoptosis was speculated to be essential for eye maturation (Meier et al., 2000; Brachmann and Cagan, 2003). Caspase dependent cell death and activation of caspase-3 was observed in chick retina (Mayordomo et al., 2003), caspase-3 deficient zebrafish showed degeneration of retina (Yamashita et al., 2008). In mice, caspase activation was shown to participate in regulation of lens transparency (Zandy et al., 2005). Similar effects were suggested to occur in chicken (Sanders and Parker, 2003). Although, caspase-3-deficient mice exhibited cataracts (Zandy et al., 2005), caspase-3 deficient lens in zebrafish remains normal (Yamashita et al., 2008).

Other evolutionary similarities in usage of caspases applies to salivary glands. Caspases regulate shaping of salivary glands in mammals (Teshima et al., 2016b). In *Drosophila*, the salivary gland is sculpted by caspase-mediated programmed cell death (Takemoto et al., 2007).

## Concluding remarks

**FIGURE 3:**
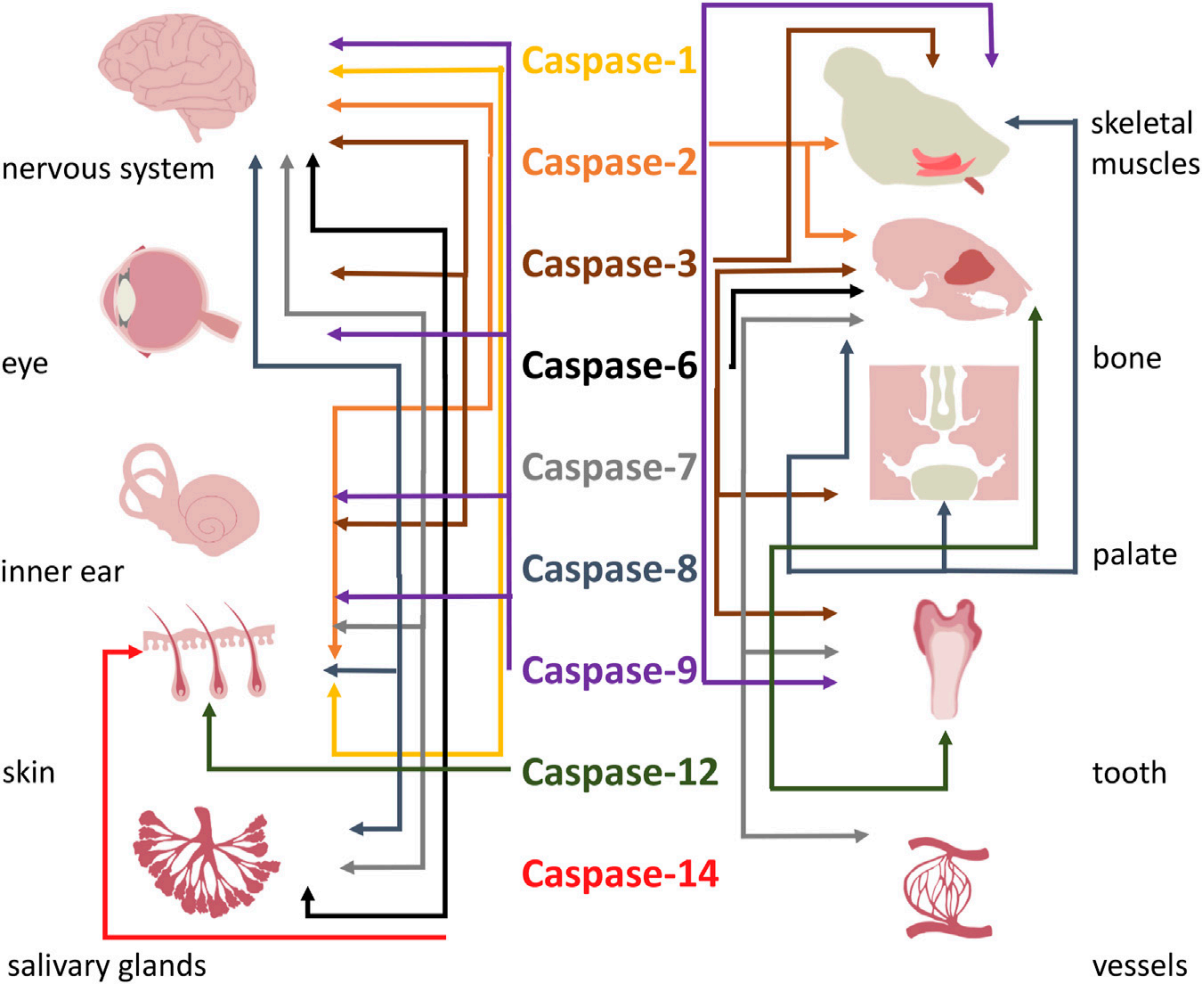
Overall functions of caspases in formation of head.

This review emphasized the importance of classical and emerging functions of caspases in development of head structures. Participation of caspases and their functions in development of the head is summarised in Table 4 and Figure 3. Caspases being assigned as apoptotic are recently considered as factors with multiple roles in many organs (Shalini et al., 2015). Their functional spectrum includes the switch between lethal and non-lethal fate of cells. The mechanisms however remain elusive. The regulation of apoptotic vs. non-apoptotic pathways may reside in the subcellular localisation of caspases (Prokhorova et al., 2018), availability of specific substrates (Nakajima and Kuranaga, 2017), compensation by anti-apoptotic proteins (Huesmann and Clayton, 2006; Grabow et al., 2018), or various levels of activation (Basu et al., 2012). Additionally, the mechanism/s might be specific in individual tissues/cell types.

**TABLE 4:** Overview of participation of caspases and their functions in development of the head region/structures.


Caspase-1	
•Decreased hippocampal neurogenesis in aging	Gemma et al. (2007)
•Impact on cell proliferation in different niches of the skin	Lee et al. (2015)
•Localised in apoptotic cells of hair follicles	Soma et al. (1998)
Caspase-2	
•Neural differentiation	Pistritto et al. (2012)
•Protection of neurons	Bergeron et al. (1998)
•Differentiation of skeletal muscles	Boonstra et al. (2018), Dehkordi et al. (2020)
•Osteoblast differentiation	Mogi and Togari (2003)
•Regulation of osteoclast apoptosis in aging	Sharma et al. (2014)
•Stimulation of osteogenic expression in calvarial osteoblasts	Kratochvílová et al. (2020)
•Regulation of hair growth	Zhang et al. (2007)
Caspase-3	
•Major actor of neuronal apoptosis	Kuida et al. (1996)
•Neural stem cell differentiation	Fernando et al. (2005)
•Reorganisation of neural cytoskeleton	Mashima et al. (1997)
•Regulation of glial cells population	Lossi et al. (2018)
•Differentiation of glial cells	Oomman et al. (2006)
•Regulation of synaptic plasticity	Chan and Mattson (1999)
•Modulation of memory	Dash et al. (2000), Huesmann and Clayton (2006), Zhuravin et al. (2015)
•Axon branching and arborisation	Campbell and Okamoto (2013)
•Axon pruning	Gu et al. (2017), Cusack et al. (2013)
•Apoptosis in the inner nuclear layer of retina	Zeiss et al. (2004)
•Protein turnover of lens cell fibres	Talebizadeh et al. (2015)
•Proteolytic cleavage of Cx45.6 associated with lens development	Yin et al. (2001)
•Regulation of lens transparency	Zandy et al. (2005)
•Normal development and function of the cochlear vestibule	Makishima et al. (2011)
•Survival of ganglion cells and hair cells	Morishita et al. (2001)
•Elimination of supporting cell in organ of Corti	Takahashi et al. (2001)
•Putative role in apoptosis of aging myocytes of extrinsic tongue muscle	Kletzien et al. (2018)
•Differentiation of rhabdomyocytes; transition of myoblasts into myotubes	Fernando et al. (2002)
•Catabolic degradation of muscular proteins	Du et al. (2004)
•Reduction of satellite cells self-renewal	Dick et al. (2015)
•Apoptotic elimination of bone-related cells in mandibular bone	Svandova et al. (2018)
•Bone ossification	Miura et al. (2004)
•Osteoblast differentiation	Mogi and Togari (2003)
•Participation in the fusion of the palatal shelves	Huang et al. (2011)
•Apoptosis in primary enamel knot	Matalova et al. (2006)
•Apoptosis accompanying formation of salivary glands	Teshima et al. (2016b)
•Apoptosis in prenatal skin	Vesela et al. (2015)
•Apoptosis of hair follicle	Soma et al. (1998), Vesela et al. (2015)
•Degradation of extracellular matrix during blood vessel remodelling	Cohen (1997), Cowan et al. (2005)
Caspase-6	
•Participation in axon pruning	Nikolaev et al. (2009), Cusack et al. (2013)
•Induction of Phex expression in calvarial osteoblasts	Kratochvílová et al. (2020)
•Putative contribution in formation of ducts of salivary glands	Teshima et al. (2016b)
Caspase-7	
•Caspase-3 redundancy in neuronal apoptosis	Houde et al. (2004)
•Regulation of osteogenic process in mandible	Svandova et al. (2014)
•Apoptotic elimination of bone-related cells in mandibular bone	Svandova et al. (2018)
•Putative participation in apoptosis of PEK	Matalova et al. (2012)
•Mineralisation of incisor enamel	Matalova et al. (2013)
•Potential participation in apoptosis during formation of salivary glands	Teshima et al. (2016a)
•Non-apoptotic regulation of mast cells population in dermis	Vesela et al. (2015)
•Potential participation in apoptosis during development of hair follicles	Vesela et al. (2015)
•Degradation of extracellular matrix during blood vessel remodelling	Cowan et al. (2005)
Caspase-8	
•Apoptosis in formation neural tube	Varfolomeev et al. (1998), Sakamaki et al. (2002)
•Potential role in differentiation of skeletal myoblasts	Fernando et al. (2002)
•Potential engagement in palatal fusion	Huang et al. (2011)
•Osteoblast differentiation	Mogi and Togari (2003)
•Apoptotic elimination of bone-related cells in mandibular bone	Svandova et al. (2018)
•Regulation of osteogenic expression	Kratochvílová et al. (2020)
•Skin homeostasis	Kovalenko et al. (2009)
•Formation of blood vessels	Varfolomeev et al. (1998)
Caspase-9	
•Neuronal apoptosis	Hakem et al. (1998), Kuida et al. (1998)
•Putative role in neural differentiation	Pistritto et al. (2012)
•Axon-selective degeneration	Cusack et al. (2013)
•Putative role in axon guidance	Ohsawa et al. (2010)
•Participation in neural tube closure	Geelen and Langman (1977), Weil et al. (1997), Hakem et al. (1998), Kuida et al. (1998)
•Apoptosis in development of retina	Laguna et al. (2008)
•Participation in elimination of nucleus from lens fibres	Sanders and Parker (2002)
•Apoptosis in vestibular organs	Cecconi et al. (2004)
•Fusion of myoblasts	Murray et al. (2008)
•Regulation of apoptosis in primary enamel knot	Setkova et al. (2007)
•Apoptosis of hair follicle stem cells, regulation of hair follicle regeneration	Ankawa et al. (2021)
Caspase-12	
•Regulation of osteogenic expression, unspecified role in differentiation of osteoblasts	Vesela et al. (2020)
•Potential function in ameloblasts and odontoblasts	Vesela et al. (2020)
•Unspecified role in development of hair follicles	Veselá and Matalová (2015)
Caspase-14	
•Terminal differentiation of keratinocytes	Lippens et al. (2000)
•Skin barrier formation	Hu et al. (1998), Hoste et al. (2011)

Thus, despite apoptosis and related molecules being investigated for half a century, the understanding of the relevant networks and their specific roles is far from complete. The expanding spectrum of functions of caspases opens many challenging questions to be addressed in the future.
