# Supplementary material for: Making the head: Caspases in life and death
Source: Front Cell Dev Biol. 2023 Jan 13;10:1075751. doi: 10.3389/fcell.2022.1075751 (PMC9880857; doi:10.3389/fcell.2022.1075751)
Supplement: Supplementary file 1 [file Table1.DOCX]

| **Apoptosis-related deficient mice** | | | |
| --- | --- | --- | --- |
| genotype | developmental defect* | genetic background |  |
| ***Caspase-2 -****/****-*** | increased dell death of motor neurons | C57BL/6J × DBA2 F | Bergeron et al., 1998 |
| ***Caspase-3 -/-*** | neural defects/premature lethality | 129X1/SvJ | Kuida et al., 1996 |
|  | delayed development of the skull | 129X1/SvJ | Miura et al., 2004 |
| ***Caspase-3 -/-*** | minimal brain defect | C57BL/6J | Leonard et al., 2002 |
|  | lens cataract | C57BL/6J | Zandy et al., 2005 |
|  | inner ear dysfunction | C57BL/6J | Makishima et al., 2011 |
| ***Caspase-3 -/-*** | dysplasia and retarded apoptosis of retina, delayed regression of vitreal vasculature | C57BL/6J-129sv  C57BL/6J-129sv/C3H | Zeiss et al., 2004 |
| ***Caspase-7 -/-*** | increased number of mast cells | C57BL/6J | Vesela et al., 2015 |
|  | osteogenic abnormalities, decreased mineralisation of enamel | C57BL/6 | Svandova et al., 2014; Matalova et al., 2012; 2013 |
| ***Caspase-8*** *-/-* | neural tube formation/embryonic lethality | C57BL/6 | Varfolomeev et al., 1998 |
| **Caspase-8^+/−/BAC-C362S^** | chronic inflammatory skin disorder | C57BL/6J |  |
| ***Caspase-9 -/-*** | neural defects/embryonic lethality | 129/C57BL/6  129/CD1 | Hakem et al., 1998 |
|  | inner ear morphogenic defect and retarded growth | C57BL/6 | Cecconi et al., 2004 |
| ***Caspase-14 -/-*** | disrupted terminal differentiation of keratinocytes | C57BL/6 | Lippens et al., 2000 |
| ***Apaf-1 -/-*** | neural defect/embryonic lethality | 129/Sv/NMRI | Cecconi et al., 1998 |
|  | inner ear morphogenic defect and retarded growth | C57BL/6 | Cecconi et al., 2004 |
|  | lack of adherence in palatal shelves, abnormal thickness of retina/perinatal lethality | C57BL/6J/129 SvEv | Honarpour et al., 2000 |
|  | defect in palatal formation | 129/Sv/NMRI | Cecconi et al., 1998 |
| ***Bax -/-*** | minor neural defects | C57BL/6 | Jung et al., 2008 |
| ***Bcl2-/-*** | progressive degeneration of facial motoneurons | C57BL/6 | Michaelidis et al., 1996 |
|  | defect in redox-regulated melanin synthesis | C57BL/6J | Veis et al., 1993 |

Supp. 1.: Genetic backgrounds used for apoptosis-related deficient mice.*This table only summarises developmental defect of the head.
